# Treatment of Erysipelas

**Published:** 1875-04

**Authors:** 


### TREATMENT OF ERYSIPELAS.

Dr. Satterlee, in the *New York Med. Jour.;* advises the quinine and opium treatment. "It consists in the administration of one, two or three full doses of the sulphate of quinine combined with enough tincture or elixir of opium to moderate the disagreeable effects of the quinine upon the head, and to assist sleep. If called at the beginning of an attack of erysipelas, I administer to an adult twenty-five to thirty grains of the sulphate of quinine, dissolved in one and a half ounces of water, which is readily accomplished by the addition of a little dilute sulphuric acid; a few drops will completely dissolve the powder, and a clear solution will be formed: to this I add fifteen minims of McMunn's elixir of opium, and we have a draught which, although very bitter to the taste, is not so disagreeable to take as a small powder of quinine; in fact, I have on one occasion administered sixty grains of quinine dissolved in three ounces of water, in one dose, to a patient with a very obstinate and long-standing intermittent fever, and the remark he made to me some time afterward was, that 'he was so glad that I had given him that draught *instead of quinine*, as he had taken a great many quinine-powders for over two years, and they were unpleasant to take, without doing him much good.' Having ordered a draught, as just stated, containing twenty-five to thirty grains of the sulphate of quinine, direct the erysipelas patient to take it all at once on retiring for the night. It will usually be retained by the stomach without difficulty; if, however, the stomach is irritable, I prescribe a mustard-plaster about the size of the hand, to be applied, ten or fifteen minutes before taking the dose, under the left breast; this procedure I have found unfailing in quieting the stomach so that the draught is retained. In one case, when the fauces were greatly inflamed and deglutition very painful, I had an equally good effect by administering the dose by the rectum. After this draught, the patient usually has a very good night, sleeping well and perspiring freely; and on examination after twenty-four hours, we find the temperature and pulse have fallen greatly. The general symptoms have either disappeared or been much improved. In some cases we have deafness and noise in the head from quinine, but in the majority of instances the opium seems to remove entirely the after-effects of the drug. The eruption markedly diminishes, and I have seen many cases where a single draught has completely aborted the disease. In all cases I direct the patient to observe simple hygienic rules, use a stimulating diet, with free draughts of lemonade where there is biliousness, a simple cathartic in constipation, and no external application whatever.

This is my treatment in the incipiency of a mild attack of erysipelas. But in any and all the varieties and severer forms of the disease, or when I do not see the case until it has advanced several days, I commence treatment in the same manner; but, at the end of twenty-four hours, or on the second evening of my attendance, I administer a second quinine draught, and, if necessary, a third at the end of forty-eight hours. In my experience this has been entirely successful in the most severe types of the disease, the eruption and general symptoms passing away with rapidity. The patient makes an excellent recovery under this mode of treatment, the appetite comes speedily, and there is very little debility experienced. Twenty-four, or at most forty-eight, hours is all that is required to abort the disease by this treatment. Having used it for three years in a large number of cases, I have never found any disagreeable after-effects; on the contrary, the general health of the patient is improved, and this is the experience of all those whom I have known, who have employed this plan.—*St. Louis Med. and Surg. Jour.*

